# Age and gender as determinants of the bone quality of the greater tuberosity: A HR-pQCT cadaver study

**DOI:** 10.1186/1471-2474-13-221

**Published:** 2012-11-13

**Authors:** Chlodwig Kirchhoff, Volker Braunstein, Stefan Milz, Christoph M Sprecher, Sonja Kirchhoff, Mathias Graw, Andreas B Imhoff, Stefan Hinterwimmer

**Affiliations:** 1 Department of Trauma Surgery, Klinikum rechts der Isar, Technische Universitaet Muenchen, Ismaningerstrasse 22, D-81675, Munich, Germany; 2 Department of Surgery-Campus Innenstadt, Ludwig-Maximilians-Universitaet Muenchen, Nussbaumstrasse 20, D-80336, Munich, Germany; 3 AO Research Institute, AO Foundation, Clavadelerstrasse 8, CH-7270, Davos, Switzerland; 4 Department of Anatomy, Ludwig-Maximilians-Universitaet Muenchen, Pettenkoferstrasse 11, D-80336, Munich, Germany; 5 Institute of Clinical Radiology - Campus Innenstadt, Ludwig-Maximilians-Universitaet Muenchen, Nussbaumstrasse 20, D-80336, Munich, Germany; 6 Institute of Forensic Medicine - Campus Innenstadt, Ludwig-Maximilians-Universitaet Muenchen, Nussbaumstrasse 7, D-80336, Munich, Germany; 7 Department of Orthopedic Surgery and Orthopedic Sports Surgery, Klinikum rechts der Isar, Technische Universitaet Muenchen, Ismaningerstrasse 22, D-81675, Munich, Germany

**Keywords:** Aging, Humeral head, Microarchitecture, Rotator cuff, Gender

## Abstract

**Background:**

Age-dependent trabecular changes of the humeral head might weaken the fixation of suture anchors used for rotator cuff (RC) repair. This might lead to suture anchor loosening and thus compromise the integrity of the repair. The aim of this study was to analyze whether the trabecular microstructure within the RC footprint is influenced by age, gender or handedness.

**Methods:**

Axial HR-pQCT scans (Scanco Medical) of 64 freshly frozen cadaveric human humeral head specimens (age 72.3 ± 17.4 years) were analyzed to determine the bone volume-to-total volume ratio (BV/TV), trabecular thickness (Trab Th), trabecular number (Trab N) and connectivity density (Conn Dens). Within the RC footprint, 2 volumes of interest (VOI), posteromedial (PM) and anterolateral (AL) and one control VOI in the subarticular bone (SC) were set.

**Results:**

The highest BV/TV was found in SC: 0.22 ± 0.06% vs. PM: 0.04 ± 0.05% vs. AL: 0.02 ± 0.04%; p < 0.05. Trab Th accounted for 0.26 ± 0.05 μm in SC, 0.23 ± 0.09 μm in AL and 0.21 ± 0.05 μm in PM. In parallel, Trab N and Conn Dens were found to be the highest in SC. Gender analysis yielded higher values for BV/TV, Trab Th, Trab N and Conn Dens for PM in males compared to females (p < 0.05). There were no significant findings when comparing both sides. We furthermore found a strong inverse correlation between age and BV/TV, which was more pronounced in the female specimens (r = −0.72, p < 0.00001).

**Conclusions:**

The presented microarchitectural data allow for future subtle biomechanical testing comprising knowledge on age- and sex-related changes of the tuberosities of the humeral head. Furthermore, the insights on the trabecular structure of the humeral head of the elderly may lead to the development of new fixation materials in bone with inferior bone quality.

## Background

Disorders of the rotator cuff (RC) are a common source of shoulder pain and disability especially in the elderly population [1]. RC tears are highly prevalent in patients aged between 55 and 70, a population with a high prevalence of osteopenia and osteoporosis [2,3]. The greater tuberosity (GT) serves as the anatomic insertion for the postero-superior RC muscles [4]. Poor bone quality and osteoporotic bone changes may compromise the success of repairs, resulting in suture anchor loosening, impaired tendon healing, and re-rupture of the RC [5,6]. Regarding the repair of such tears, Barber et al. demonstrated a decreased pullout strength of suture anchors in the anterior compared to the posterior part of the GT [7]. The authors hypothesized that this mechanical difference might be related with regional bone quality differences. In this context, Yakacki et al. analyzed seven humeral heads using high-resolution peripheral quantitative computed tomography (HR-pQCT) and gave a first subtle hint on the correlation of osseous microarchitectural patterns and anchor pullout strength [8]. Amendatory, significant regional differences of Bone Mineral Density (BMD) within the GT have been recently reported in a larger series of osteoporotic humeral heads [9]. Although precise information on the microarchitecture of the humeral head might contribute to future anchor designs, detailed information on regional differences in bone quality parameters related to patients’ age, gender and handedness is still lacking.

Therefore, the aim of the present study was firstly to assess the bone structure parameters trabecular thickness (Trab Th), trabecular number (Trab N) and connectivity density (Conn Dens) and secondly to determine whether humeral bone structure changes with age, whether there are sex-related differences, and if there are variations between the dominant and the non-dominant shoulder.

## Methods

### Specimen preparation

Sixty-four paired human humeri from thirty-two human cadavers without signs of posttraumatic changes, previous surgical interventions or tumor lesions were freshly harvested and stored at −20 degrees C. All specimens originated from the local Institute of Forensic Medicine. The age ranged from 38 to 98 years (72.3 ± 17.4 yrs.), there were 15 males and 17 females. There was no statistical difference regarding age between both gender groups. After thawing to room temperature, the shoulders were inspected to confirm an intact rotator cuff and stripped of all soft tissue for a clear determination of the anatomy. Biplane radiographs were performed to detect any bony abnormalities. Shoulders with signs of posttraumatic changes, previous surgical intervention or tumor lesions were excluded from the study. Information regarding handedness was acquired from the next of kin. The study was approved by the local ethics committee (reference number 47–09).

### High resolution quantitative computed tomography (HR-pQCT)

**Figure 1:**
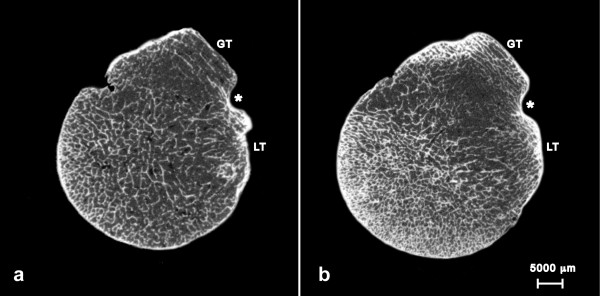
**Representative axial HR-pQCT scans of the humeral head.a**) depicts the cross section of a left humeral head of a female patient (74 years) with inferior bone quality within the greater tuberosity (GT). **b**) depicts a section of the same height of a left humeral head of a male patient (65 years) with normal bone quality within the greater tuberosity (GT). For orientation the bicipital groove is labeled with *, the lesser tuberosity with LT.

For the scanning procedure the proximal humerus was fixed horizontally with the lesser tuberosity in a 12-o’clock position. A horizontal line running through the lowest point of the articular surface defined the inferior border of the humeral head. The HR-pQCT imaging system (XtremeCT, Scanco Medical, Brüttisellen, Switzerland) is equipped with a 70 μm focal spot. The X-ray tube was operated at 60 kVp and 900 μA. The integration time was set to 300 ms. Two-dimensional CT images were reconstructed in 1536 by 1536 pixel matrices from 750 projections using a standard convolution-backprojection procedure (see Figure 1). Images were stored in 3D arrays with an isotropic voxel size of 82 μm. The complete proximal end of the humerus was acquired with a total of 550–600 microtomographic slices, corresponding to a length of 45–50 mm. All HR-pQCT scans were performed and analyzed by the same investigator (CK).

### Image processing and evaluation of bone quality

A Gaussian filter with a sigma of 0.7 and support of one voxel was primarily used to suppress noise. A uniform threshold of 11% of the maximal gray scale value was selected for all samples for binary segmentation, based on the manufacturer's software. This value represents the peak of bone tissue in the gray value distribution histogram.

**Figure 2:**
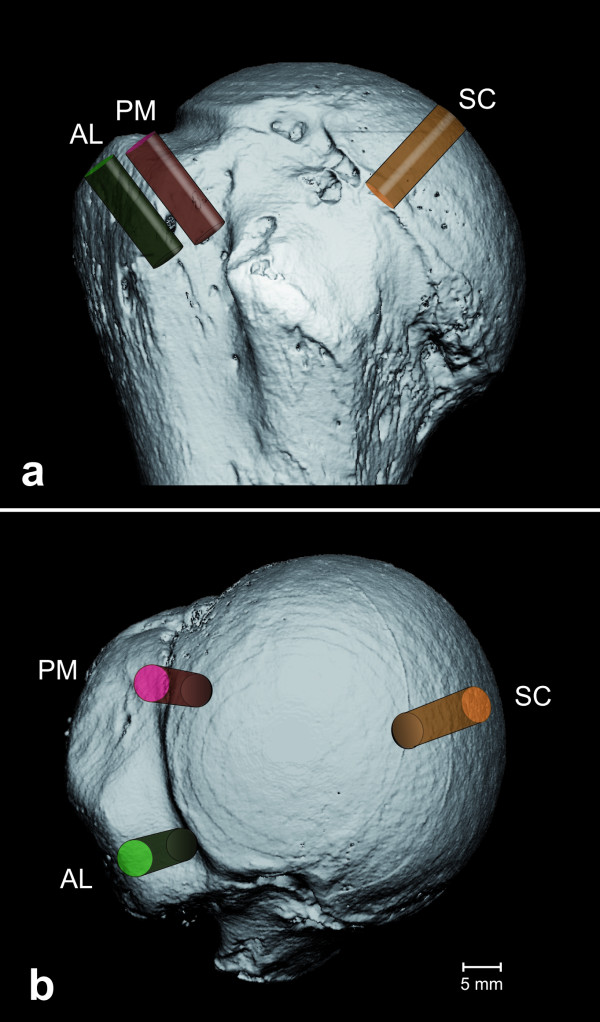
**Placement of the regions of interest.a**) depicts an ap-view of the lesser tuberosity. Within the greater tuberosity, two rows were defined; one medial row adjacent to the articular surface, labeled with PM; and one lateral row along the lateral edge of the footprint, labeled with AL. Furthermore, a region was set into the subchondral region directly underneath the articular surface, marked with SC. **b**) gives an axial view of the humeral head with the lesser tuberosity on the bottom. The GT is divided into one posteromedial portion (PM) and one anterolateral part (AL).

For defining the volumes of interest (VOIs) for further analysis, the borders of the footprint within the GT were defined in each specimen. Consecutively, the GT was divided into three equally-sized quadrants. Furthermore, two rows were defined; one medial row, directly adjacent to the articular surface and one lateral row along the lateral edge of the footprint. Subsequently, two VOIs were defined; one was set into the posterior quadrant of the medial row (PM), and one into the anterior quadrant of the lateral row (AL). Furthermore, a VOI (SC) was set directly underneath the articular surface serving as a control region (for volume positions see Figure 2). Each VOI had a cylindrical shape with a diameter of 7.5 mm and a depth of 18 mm corresponding to the average volume of suture anchors currently used for RC repair [10]. Each VOI was placed at a 45° angle to the GT [11]. The segmented VOIs contained only cancellous bone. The bone volume (BV) was calculated using a tetrahedron meshing technique generated with the marching cubes method. The total volume (TV) was identical to the cylindrical VOI. From BV and TV, the mean bone volume to total volume (BV/TV) was derived. In addition, mean Trab Th [μm] and Trab N [μm^-1^ were calculated directly using a model-independent sphere-filling technique. Conn Dens [μm^-3^ was calculated based on the Euler number. Image processing and analysis were performed using the software package provided by the scanner’s manufacturer (Image Processing Language, Scanco Medical, Brüttisellen, Switzerland).

### Statistical analysis

Data are given as mean ± standard deviation (mean ± SD). The results of the different VOIs regarding BV/TV, Trab Th, Trab N and Conn Dens were compared using the Mann–Whitney-*U*-test. For assessing differences of BV/TV, Trab Th, Trab N and Conn Dens between males and females, a parametric analysis of variance (ANOVA-on-ranks) followed by the post-hoc Dunn’s test was applied. For the detection of differences between the dominant and the non-dominant side regarding BV/TV, Trab Th, Trab N and Conn Dens, ANOVA-on-ranks was calculated as well. For analysis of the correlation between patients’ age and BV/TV, Trab Th, Trab N and Conn Dens the calculation of Spearman rank order was performed, as the variables were not normally distributed. The level of significance was set at p < 0.05 or <0.001 respectively. Statistical analysis was performed using the Sigma Stat 3.5 software (Systat Inc, Chicago, IL, USA).

## Results

### General morphology

Comparing the three different sectors, the highest BV/TV was found in the subchondral region (SC: 0.22 ± 0.06%, PM: 0.04 ± 0.05%, AL: 0.02 ± 0.04%; p < 0.05). Regarding Trab Th, the highest thickness was also found in sector SC (0.26 ± 0.05 μm), followed by sector PM (0.21 ± 0.05 μm) and AL (0.23 ± 0.09 μm). A statistically significant difference was detected between SC and PM (p < 0.05). Trab N in sector SC was the highest and differed significantly from the values for PM and AL (SC: 1.42 ± 0.61 μm^-1^ vs. PM: 0.54 ± 0.25 μm^-1^ vs. AL: 0.32 ± 0.10 μm^-1^).

**Figure 3:**
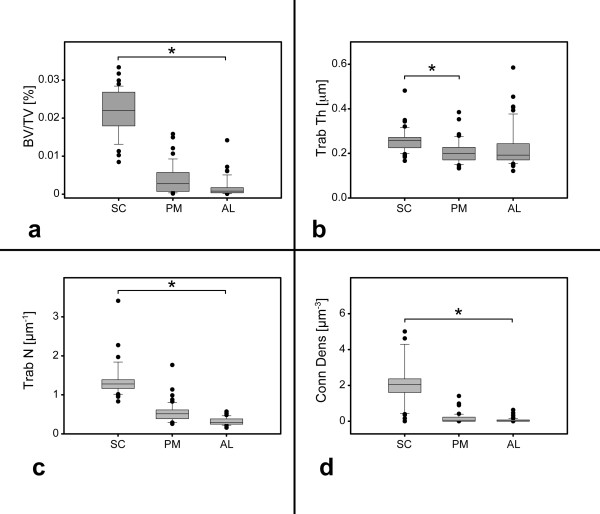
**Morphometric parameters of bone quality.a**) the ratio bone volume/total volume (BV/TV) is given as percentage [%]. Data for the subchondral region are labeled as SC, followed by the posteromedial portion PM and the anterolateral AL. **b**) presents data for trabecular thickness (Trab Th) in μm. **c**) presents data for trabecular number (Trab N), given in [μm^-1^]. In **d**) data for connectivity density (Conn Dens) are presented in [μm^-3^]. Data are given as boxplots, * p < 0.05.

In parallel, Conn Dens was also found to be significantly higher in SC (2.33 ± 1.87 μm^-3^) compared to PM (0.18 ± 0.31 μm^-3^) and AL (0.06 ± 0.12 μm^-3^). Detailed information is presented in Figure 3.

### Gender analysis

**Figure 4:**
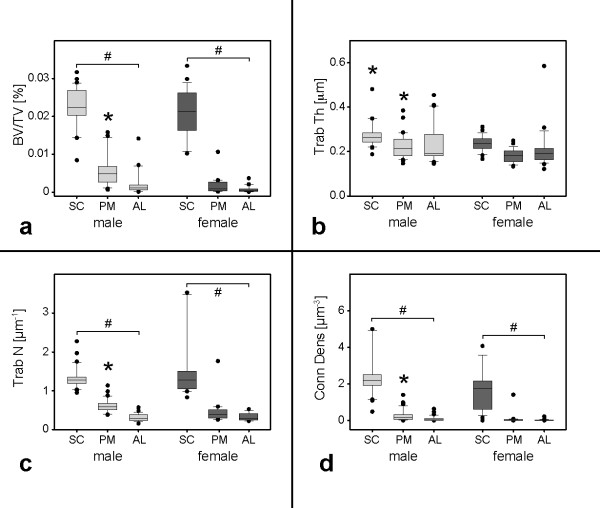
**Gender analysis.** Fig. **a**) presents results for BV/TV in [%], **b**) for Trab Th in [μm], **c**) for Trab N in [μm^-1^] and **d**) for Conn Dens in [μm^-3^]. Data for SC, PM and AL in males are depicted in light grey, for females in dark grey. Data are given as boxplots. # indicates differences within each gender group (p > 0.05). * marks inter-gender differences (p < 0.05).

The analysis regarding gender differences revealed distinct findings. BV/TV in sector SC of males accounted for 0.22 ± 0.05% vs. 0.21 ± 0.06% in females. A significant difference was found comparing BV/TV of sector PM (males: 0.06 ± 0.05%, females: 0.02 ± 0.02%; p < 0.05). In contrast, the values for AL did not show any significant difference between both genders (male 0.03 ± 0.05% vs. female 0.01 ± 0.01%). In males as well as in females SC, PM and AL were significantly different with the highest BV/TV values found in SC, followed by PM and AL (p < 0.05). Trab Th of sector SC revealed no differences comparing genders (males: 0.27 ± 0.05 μm, females: 0.24 ± 0.04 μm). In parallel, no significant difference was found in sector PM (males: 0.22 ± 0.06 μm vs. 0.18 ± 0.03 μm) and AL (males: 0.24 ± 0.09 μm vs. 0.1 ± 0.09 μm). Though SC always showed the highest Trab N in both genders, PM always revealed the lowest (p < 0.05). Trab N yielded significantly higher values in males in comparison to females (0.61 ± 0.28 μm^-1^; 0.46 ± 0.29 μm^-1^; p < 0.05). Again, the highest values were found in SC. In accordance to BV/TV, Trab Th and Trab N, the Conn Dens resulted in significantly higher values in the male samples (0.26 ± 0.31 μm^-3^ vs. 0.08 ± 0.28 μm^-3^). The highest values were again found in sector SC, followed by PM and AL (see Figure 4).

### Handedness analysis

**Figure 5:**
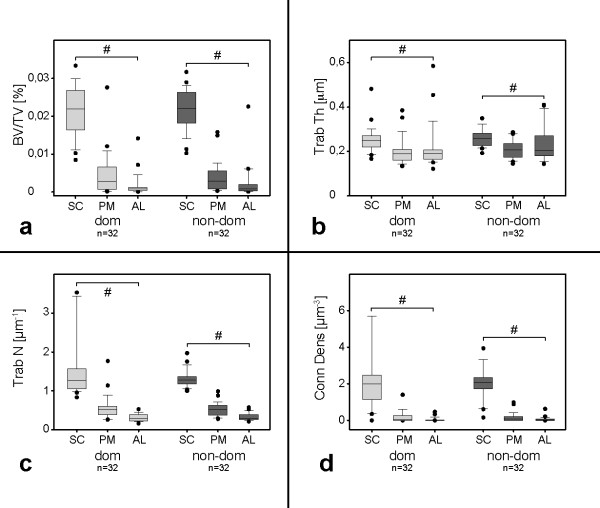
**Handedness analysis.** Fig. **a**) presents results for BV/TV in [%], **b**) for Trab Th in [μm], **c**) for Trab N in [μm^-1^] and **d**) for Conn Dens in [μm^-3^]. Data for SC, PM and AL for the dominant (dom) side are depicted in light grey, for the non-dominant (non-dom) side in dark grey. Data are given as boxplots. # marks intra-group differences (p < 0.05).

Relating the analysis of differences between the dominant and the non-dominant humeral head, no statistically significant findings resulted (see Figure 5).

### Age correlation

**Figure 6:**
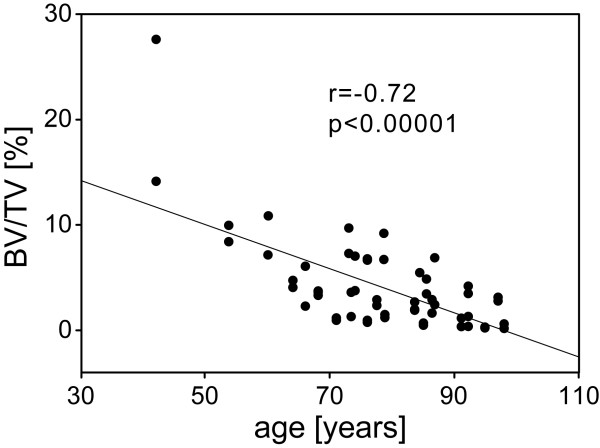
**Linear regression analysis correlating the results of HR-pQCT regarding the ratio bone volume/total volume (BV/TV) within AL and patients’ age (years).** A correlation of −0.72 was found (p < 0.00001).

In a final step, the data were correlated to the patients’ age. A strong inverse correlation with a correlation coefficient of −0.72 was found for BV/TV of sector AL (p < 0.00001). Regarding BV/TV of PM as well as of SC, an inverse correlation was found. However, this trend was not statistically significant. In comparison of the genders, this inverse correlation between age and BV/TV in terms of a bone quality parameter was more pronounced for the female specimens reaching significance (p < 0.05). In addition, an inverse correlation between Trab N and age was also found for the female specimens (p < 0.001). However, no correlation could be detected for Trab Th and Conn Dens and age (see Figure 6).

## Discussion

Clear information regarding microarchitecture of the GT might influence the choice of anchoring techniques and implant positioning. Although the success of RC surgery is majorly determined by the stability of the suture tendon interface and the footprint restoration, evidence grows in the very recent past that the security of anchorage has been overestimated [12-15]. Therefore, poor bone quality may compromise the success of RC repair by promotion of suture anchor loosening and thereby impaired tendon healing [6,9,14].

Therefore, the trabecular architecture of the GT of osteoporotic humeral heads using HR-pQCT was analyzed in the present study. In accordance with previous work, this study also revealed higher values of bone volume to total volume ratio (BV/TV) in the posteromedial GT-region in comparison to the anterolateral portion [9]. In addition, further crucial bone quality parameters such as trabecular number (Trab N), trabecular thickness (Trab Th) as well as connectivity density (Conn Dens) were assessed in the presented work resulting in a distinct pattern regarding the regional distribution within the GT. In comparison between both genders, the posterior quadrant of the GT, directly adjacent to the articular surface, appeared to be of even better bone quality in males. Regarding the comparison between the dominant and non-dominant shoulder, no significant differences for any bone quality parameter resulted. Moreover, a distinct inverse correlation of BV/TV and age was found with a more pronounced character in the female specimens.

In the present study, a HR-pQCT imaging system from Scanco Medical, Switzerland, was used with an isotropic voxel size of 82 μm providing a direct analysis of BV/TV, Trab N, Trab Th and Conn Dens. HR-pQCT is a non-destructive technique allowing for measurements of important micro-architectural parameters of bone morphology. Several authors clearly demonstrated the biomechanical consequence of the assessed parameters [16-19]. In this context, Schiuma et al. investigated the primary stability of proximal humeral nails (MultiLoc PHN) in correlation to bone properties assessed by HR-pQCT. The authors found a significant correlation between axial displacement and bone properties at the primary proximal screws [17].

There are several studies in the literature trying to characterize the bone architecture of the GT [5,7,20,21]. However, most of these studies suffer from significant technical drawbacks such as Barber et al., who were trying to correlate biomechanical data for anchor pullout with BMD of the GT, assessed by dual-energy X-ray absorptiometry (DXA) [7]. Although they found higher pullout forces in the posterior GT part, they failed to show any significant differences between the anterior and posterior GT areas because of the DXA technique used and the consecutively impossible measurement of microarchitecture that only evaluated bone mineral density [7]. On the other hand, Tingart et al. performed pQCT-scans with a pixel size of 0.59 mm and a slice thickness of 2.5 mm, thereby acquiring scans of inferior resolution [5].

One of the relevant characteristics of bone quality is its microarchitecture, which is influenced by bone turnover and mineralization. Lill et al. reported a correlation of increasing age and decreasing BMD [22]. In contrast to our results, they found the highest BMD in the medial and posterior regions of the cranial horizontal slice in women. They also stated that a BMD decrease for women older than 70 years is constant over all regions of the proximal humerus. In this context, our data are more in line with Barvencik et al. reporting an accentuation of bone loss in the lateral humeral head [23]. In our study, BV/TV was higher in the medial compared to the lateral region, independent from age and sex. This is in accordance with the work of Tingart et al., however they used a pQCT with inferior resolution and thus their results are not really comparable [5]. Khosla et al. stated that the microstructural basis for the trabecular volume differs between the sexes whereas the BV/TV declined similarly in men and women with advancing age [24]. Regarding the number of trabeculae, the authors reported a decrease of Trab N with a corresponding increase of Trab Sep in terms of a trabecular loss in women, whereas the primary mechanism for the decrease of BV/TV is found in trabecular thinning in men. These facts have a significant impact on the age-related bone strength, since based on a finite element, modeling a decrease in Trab N has a 2- to 5-fold greater impact on bone strength in comparison with Trab Th reduction resulting in a similar decrease in bone volume [25]. Our results are in line with these findings, as for our rather old (mean age 72 yrs) specimens a significantly higher BV/TV and Trab N was found for the males, whereas no significant difference was found for Trab Th between both genders. Although the trabecular microarchitecture of the humeral head was assessed non-invasively using HR-pQCT, the presented results in female specimens are consistent with the work of Parfitt et al., who analyzed transiliac biopsies of pre- and postmenopausal women using histomorphometry [26]. Similarly to our data, they showed that postmenopausal women had significantly reduced BV/TV along with reduced Trab N and correspondingly increased Trab Sep. These facts formed the basis for the possible hypothesis that a menopause-associated increase in osteoclastic activity leads to a loss of all trabeculae with a corresponding conversion of the continuous trabecular network characteristic for young women into the discontinuous network of older women [26]. Since we reported similar changes in bone quality at the humeral head using high-resolution pQCT of rather old postmenopausal females, an effect of menopause on this process cannot be excluded but seems rather probable. In contrast, there are micro-CT studies with discordant findings highlighting the need for further studies to analyze the changes of the trabecular structure in women across the menopausal transition [26]. Regarding the male specimens, these findings are consistent with those published by Aaron et al. on cadaveric transiliac bone biopsies describing a parallel decrease of BV/TV with advancing age in men and women and similar to the presented data a significant decrease of Trab N in the course of a lifetime in women, but not in men [27]. Moreover, the presented data is also consistent with the findings on Trab Th as described by Khosla et al. showing a more pronounced decrease of Trab Th at the wrist in men compared to women in the course of a lifetime [24].

Bone quality is an important factor not only for fracture management but also for the preoperative planning of RC repair, especially in the elderly population. There are currently no concepts to quantify the local degree of osteoporosis of the humeral head before surgical treatment. However, in their study Diederichs et al. reported indications for the existence of alternative BMD measurement sites in case of proximal humerus fractures using QCT, such as the contralateral side helping the physician to choose the adequate surgical procedure for treatment [28]. In their study, a high correlation of BMD values between the right and left proximal humerus resulted. Our results are in line with theirs since the comparison of bone quality parameters of the right and left humeral head assessed with HR-pQCT revealed no significant differences, neither for gender nor for any bone quality parameter, which might be due to similar biomechanical loading conditions.

With increasing age, the overall bone mass decreases, leading to a reduction in trabecular thickness and trabecular connectivity [29]. This reduction in bone mass and bone quality is predominantly seen in women older than 50 years and older in comparison to men [30]. These changes seem to be most prominent in the proximal regions of the long tubular skeletal bones such as the proximal femur, the tibial head and also in the humeral head, as shown in the study by Hepp et al. [31]. However, the results of the histomorphometric analysis of these other mentioned skeletal regions cannot be transferred directly to the situation of the humeral head. The knowledge about the total mineral density of the humeral head mostly derived from DXA is not sufficient to differentiate between regions with adequately stable bone versus bone with minor mechanical strength. This information on bony mechanical strength which is considered to be a function of age and gender is essential to achieve minimally invasive but maximally stable osteosynthetic results in case of fractures as well as stable suture anchor fixation in case of RC tear.

Amling et al. and Mosekilde et al. reported a correlation between increasing age and diminished BV/TV in females for spinal and proximal femoral specimens [30,32]. The presented analysis for BV/TV and trabecular network of the humeral head showed a relationship between age and gender as well as bone quality and quantity. In the female specimens, a significantly inverse correlation between age and BV/TV was found, whereas such an inverse correlation was not as pronounced in male specimens. This inverse correlation between the genders can be explained by a reduction in trabecular number which can be derived from the significantly inverse correlation of trabecular number with advancing age in females. Another reason might be the reduction of trabecular node number with a significantly negative correlation in females as described by Hepp et al. which is partly a contrast to our results, since we did not find any inverse correlation between Conn Dens and age for both genders [31].

One potential drawback of our study was the enrollment of shoulder specimens with a macroscopically intact RC only. Of course, this situation does not reflect the actual clinical situation and future examinations need to focus on this problem which will be part of ongoing trials of our study group.

## Conclusions

The present study comprises substantial anatomical data for future biomechanical testing of humeral heads. This knowledge of microarchitectural distribution within the humeral head regarding sex- related and age-dependent changes will allow a more sophisticated testing of areas with anticipated high bone mass against regions with expected low bone mass. This finally may lead to the development of new fixation techniques for bone of lowered quality such as in case of osteoporosis. Considering the major drawback of our study in terms of enrolling only specimens with macroscopically intact RC, it may be of interest to assess the bone quality of patients suffering from chronic RC tears.

In conclusion, it might be advantageous to consider the current findings in orthopedic surgery regarding RC repair. A placement of suture anchors within the lateral or anterior part of the GT may result in an unpredictable and probably poor mechanical stability.

## Competing interests

The authors declare that they have no competing interests.

## Authors' contributions

CK, VB, SM and CMS contributed to study design, data collection and analysis, and drafted the manuscript, SH, SK, MG and ABI contributed to study design and manuscript review. All authors read and approved the final manuscript.

## Pre-publication history

The pre-publication history for this paper can be accessed here:

http://www.biomedcentral.com/1471-2474/13/221/prepub
